# Gait Deviations in Children with Autism Spectrum Disorders: A Review

**DOI:** 10.1155/2015/741480

**Published:** 2015-04-02

**Authors:** Deirdre Kindregan, Louise Gallagher, John Gormley

**Affiliations:** ^1^Discipline of Physiotherapy, School of Medicine, Trinity College Dublin, Dublin, Ireland; ^2^Discipline of Psychiatry, School of Medicine, Trinity College Dublin, Dublin, Ireland

## Abstract

In recent years, it has become clear that children with autism spectrum disorders (ASDs) have difficulty with gross motor function and coordination, factors which influence gait. Knowledge of gait abnormalities may be useful for assessment and treatment planning. This paper reviews the literature assessing gait deviations in children with ASD. Five online databases were searched using keywords “gait” and “autism,” and 11 studies were found which examined gait in childhood ASD. Children with ASD tend to augment their walking stability with a reduced stride length, increased step width and therefore wider base of support, and increased time in the stance phase. Children with ASD have reduced range of motion at the ankle and knee during gait, with increased hip flexion. Decreased peak hip flexor and ankle plantar flexor moments in children with ASD may imply weakness around these joints, which is further exhibited by a reduction in ground reaction forces at toe-off in children with ASD. Children with ASD have altered gait patterns to healthy controls, widened base of support, and reduced range of motion. Several studies refer to cerebellar and basal ganglia involvement as the patterns described suggest alterations in those areas of the brain. Further research should compare children with ASD to other clinical groups to improve assessment and treatment planning.

## 1. Introduction

Autism is a developmental disorder which presents before three years of age [1]. It is a spectrum of pervasive developmental disorders and is found across all ethnic cultures and economic groups. From 2013, all children with autism, Asperger's syndrome, or other pervasive developmental disorders will receive one umbrella diagnosis of autism spectrum disorder (ASD) [2]. There has been a steady increase in the prevalence of ASD over the last twenty years [3, 4]. This may however be due to increased access to diagnostic services or increasing awareness of the condition. Children with ASD have difficulty with social interaction, communication, and language skills with many children demonstrating restrictive and repetitive behaviour [5, 6]. These kinds of behaviour may include rocking, finger flicking, or arm flapping [7]. Motor stereotypies are defined as “involuntary, coordinated, patterned, repetitive, rhythmic, and purposeless but seemingly purposeful movements” [8]. Children with ASD have been found to demonstrate numerous gait stereotypies such as pacing, jumping, hopping, skipping, and spinning and it has been suggested that these may also be considered restrictive and repetitive behaviour [9]. Gait abnormality can be simply defined as a deviation from normal walking pattern and may include, but is not limited to, the above mentioned stereotypies.

The “manner or style of walking” or gait is described as a method of locomotion using reciprocal placement of the lower limbs to provide both propulsion and support by Levine et al. [10]. Alterations in movement patterns in children with ASD were noted as far back as 1943 by Kanner who found that those with ASD often demonstrated “clumsy” gait and gross motor patterns [11]. In more recent years, Ghaziuddin and Butler found that children with ASD demonstrated poorer coordination than those with Asperger's disorder [12]. Many studies have subsequently examined motor coordination in children with ASD and a recent review, by Fournier et al., provided further evidence that children diagnosed with ASD may be “less coordinated and show fewer motor capabilities” [13]. This may therefore suggest that gait disturbances may be common among children with ASD. For example, it has been observed that children with ASD are more prone to idiopathic toe walking than age-matched healthy controls [14, 15]. This, however, is only evident in children under six years of age.

The motor deficits reported in association with ASD, that is, impaired vestibular control and fine and gross motor abnormalities, have been likened to patients with known cerebellar deficits [16]. Deficits in smooth pursuit and saccadic eye movements are reported in ASD and are suggestive of vermal dysfunction in the cerebellum [17, 18]. Neuroimaging studies in children with ASD show reduced ipsilateral activation of the cerebellum during gross motor movement and more diffuse activation in lobules VI-VII [19, 20]. Cerebellar deficits are widely reported in ASD, reduced Purkinje and granule cells, and vermal hypo- and hyperplasia have been reported and both pre- and postnatal processes have been implicated [21–25]. The cerebellum has been implicated in the motor deficits in ASD via connections with the parietal lobe and wide ranging connections with cortical and subcortical brain regions serve to modulate multiple brain functions that are impaired in ASD [26]. Deficits in postural control and gait in ASD have been linked to dysfunction in sensory integration to the cerebellum or to the basal ganglia due to similarities with gait abnormalities observed in Parkinson's disease [13, 27, 28]. Abnormalities in basal ganglia shape have also been associated with motor deficits in ASD [29].

In adults, the first study on kinematic and kinetic gait patterns in ASD was carried out by Hallett et al. [30]. Adults with ASD were found to demonstrate “mild clumsiness” during gait but the only significant abnormality was a reduced range of motion at the ankle joint. Following on from this, in the last twenty years, there have been several papers published which examined gait patterns in children with autism spectrum disorders. The objective of this review is to identify gait abnormalities that may be present in children with ASD. Identification of gait abnormalities may permit earlier diagnosis and better treatment planning.

## 2. Methods

### 2.1. Terminology

The terms kinematics and kinetics are used to describe gait. Kinematic analysis of gait describes the linear and angular displacement, velocities, and accelerations of motion. Inherent in kinematic analysis is the description of motion from a temporospatial perspective which describes, for example, step or stride lengths as well as cadence and gait velocity. Kinetics is the study of the forces that cause motion. In movement analysis, kinetic parameters define the forces causing the movement. The most common force acting on the body during gait is the ground reaction force (GRF), which is the force exerted by the ground on the foot [31]. Another kinetic parameter commonly described is the joint moment which is the turning effect of a force generated by a muscle across a joint.

### 2.2. Search Strategy

Articles included in this review were retrieved from five online databases (PubMed, EmBase, Medline, CINAHL, and Web of Science) by a single investigator. The following key terms were used: gait, autism. Reference lists of the articles retrieved in this search were then manually searched.

### 2.3. Selection Criteria

Studies which assessed the relationship between temporospatial, kinematic, or kinetic gait parameters and ASD in children aged between four and 18 years were selected for inclusion in this review. Studies written in English and published between January 1970 and February 2015 were included. Of 126 studies retrieved in the search, only 11 studies met the inclusion criteria and were therefore selected and included in this review.  Table 1 shows the studies included for review.

## 3. Results

### 3.1. Temporospatial Parameters

Temporal and spatial parameters refer to gait parameters which are related to timing and displacement or distance. The temporospatial parameters that have been examined in children are stride length, step length, step width, cadence (steps per minute), velocity, stance time, and double support. Of the ten studies that examined stride/step length, five found that stride length/step length was significantly reduced in children with ASD compared to healthy controls [33, 37, 39–41], which is consistent with the study by Hallett et al. who found a reduced stride length in adults with ASD. The other five studies, however, found no significant differences in stride length between children with ASD and controls [28, 34–36, 38]. Step width was assessed in four studies and has been found to be significantly increased in children with ASD in two [32, 35, 37], but no significant differences were found by another [38].

Of the 11 studies, eight assessed cadence. Calhoun et al. [36] found that children with ASD had, on average, a higher cadence than the controls, contrasting with Weiss et al. [33] who found it to be reduced in the ASD group and the six studies which found no significant difference between groups [28, 34, 35, 37–39, 41]. Velocity was assessed by nine studies, but no significant differences between children with ASD and controls were found by six of them [28, 34–36, 38, 39]. Velocity was, however, found to be significantly reduced in the ASD in two studies [33, 40] and slightly reduced in a third [37]. In two studies, stance time was found to be significantly increased [33, 41] but no significant difference was found in four other studies [34, 36, 37, 39]. Double support was examined by six studies, but only one found significant differences. Weiss et al. [33] found that double support time was significantly increased in the ASD group when compared to controls.

The wide variation in temporospatial results between studies may be due to varying inclusion criteria such as age, gender, and IQ or differing gait analysis methods.

### 3.2. Kinematic Parameters

**Figure 1:**
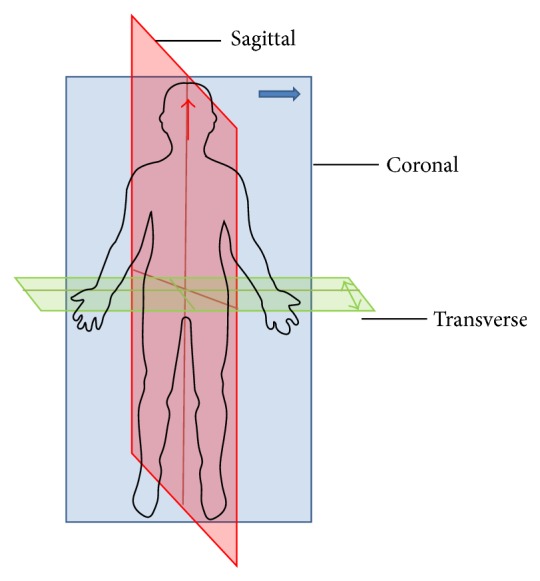
Anatomical planes of the body.

Kinematics is the study of motion without regard to the forces that cause it. In gait, kinematic parameters refer to joint motions and angles at specific points in the gait cycle. These motions occur within three planes: the sagittal plane, the coronal plane, and the transverse plane (Figure 1). The sagittal plane is the plane through which most of the motion of gait occurs. As such it is the plane that splits the human body into right and left and the plane through which forward/backward motion occurs, for example, flexion/extension of the hip. The results will be discussed by joint in order of ankle, knee, and hip.

Children with ASD were found by Vilensky et al. [41] to have reduced dorsiflexion of the ankle joint at ground contact but found other ankle joint angles to be within normal limits [41]. At toe-off, significantly reduced plantar flexion was detected by Nobile et al. in children with ASD compared to controls [37]. Children with ASD had overall reduced range of motion at the ankle joint [40]. Ambrosini et al. [40] found that children with ASD had slightly, but not significantly, increased dorsiflexion during midstance and toe-off. This may be interpreted to mean they had reduced plantar flexion at toe-off, as found by Nobile et al. [37].

At the knee, children with ASD were found to have significantly reduced ROM with a decreased flexion-extension angle at toe-off when compared to healthy controls [37]. Significantly reduced knee extension in children with ASD was also found by Vilensky et al. [41] only this time at initial contact. At the hip joint there is no consensus with Nobile et al. [37] finding a significantly reduced range of motion at the hip but Vilensky et al. [41] found that the children with ASD had increased hip flexion at toe-off.

### 3.3. Kinetic Parameters

Kinetic gait parameters are those which are concerned with the forces involved in the production of movement. Calhoun et al. [36] and Ambrosini et al. [40] are the only studies to investigate kinetic gait parameters in children with ASD. Children with ASD were found to have reduced peak plantar flexion moments at the ankle but all other ankle kinetics were within normal ranges [36]. No significant differences were found in knee joint kinetics, but one significant difference was found in hip joint kinetics between groups. The children with ASD had decreased peak hip flexor moments compared to the control group [36]. Ground reaction forces are the forces exerted by the ground on an object or body in contact with it. Children with ASD were found to have relatively normal ground reaction forces, with the exception, being the second vertical peak which was reduced in children with ASD when compared to normative data [40]. The second vertical peak refers to the ground reaction force during the period of terminal stance, which ends with toe-off.

### 3.4. Variability in Gait

Three studies investigated the effect of external factors on the variability of gait in children with ASD. One study [35] examined the effect of self-determined speed on the temporal spatial gait patterns of children with ASD. They asked the children to walk at their normal pace and then asked them to walk at faster and slower rates and found that the children with ASD widened their base of support while walking at increased speed. They also studied the effect of cueing and concurrent tasks on gait in children with ASD. Their results showed that visual cues significantly increased stride length variability in children with ASD, but effects of dual tasks, tapping while walking (motor task) and counting while walking (cognitive task), were not statistically significant. Some studies found that children with ASD had significantly increased variability in their stride lengths [28, 38], walking velocities, and stride times [38]. One study found that children with ASD demonstrated an unusual cadence-stride length relationship in their gait pattern, with an increased stride length at a given cadence compared to controls [35].

In summary, between-group gait parameter varied across the different studies. Stride/step length was found to be increased in ASD in half of the studies that examined it. Step width was assessed in two studies and was found to be increased in both. Cadence was found to be increased in children with ASD in one study but decreased in another, and no significant differences were found by six other studies. Stance time has been found to be increased in ASD in two studies, but other studies found no differences. Joint ranges of motion were found to be significantly different in children with ASD compared to controls in all studies that presented kinematic data. However the joints affected and the periods of the gait cycle in question varied between studies. Two studies assessed kinetic gait parameters. The ASD groups demonstrated altered joint moments at the ankle and the hip in one study and ground reaction forces were found to be reduced in another.

## 4. Discussion

Although there are few studies completed in the area of gait analysis in children with ASD, some emerging commonalities were identified. Some studies have found differences in temporal and spatial gait parameters between children diagnosed with ASD and healthy controls. The most common deviations found are an increased step width and, debatably, a decreased step length and stride length. Nayate et al. [35] revealed an increase in stride length at a given cadence when compared to controls, which may account for the disagreement in results between studies, as this may not have been taken into account [35]. Cadence was found to be increased in children with ASD in one study, and since the children were found to take smaller steps in many studies, this is not surprising. An increased step width gives the children a wider base of support, and reduced step and stride lengths allow them to keep their centre of gravity firmly within this base of support. This, combined with reduced velocity and increased time in the stance phase of gait, suggests a tendency to augment their stability during walking. This may be due to issues with balance [42], proprioception [43], or behavioural anxiety [44].

Of the studies in this review, three which studied kinematic parameters have found reduced range of motion during gait in children with ASD, especially ankle dorsiflexion. Nobile et al. [37] and Ambrosini et al. [40] found that there was increased ankle dorsiflexion (i.e., decreased plantar flexion) at toe-off. Vilensky et al. [41] found that children with ASD had reduced dorsiflexion at heel strike but did not find any significant differences at toe-off. It is not possible therefore to draw a conclusive picture of changes at the ankle joint during gait in children with ASD. At the knee, children with ASD were observed to have reduced ROM in two studies [37, 41], but there was no agreement at the hip joint with one study stating that children with ASD had reduced hip ROM [37] but another suggesting an increase in hip flexion in children with ASD [41]. An increase in hip flexion would fit in with the above findings of reduced plantar flexion at toe-off as increased hip flexion would compensate for the reduced propulsive force and aid in foot clearance as the ipsilateral limb enters the swing phase.

Overall, the kinematic findings of these studies are sparse and often contradict each other. There is a lack in comparability in results as analysis differs. For example, Vilensky et al. [41] concentrate on the angles at initial contact, whereas Nobile et al. [37] focus on those at toe-off. These possibly suggest that there is a general reduction in range of motion at the joints of the lower limbs in children with ASD during gait. However, little research has been done solely on joint mobility in ASD so it is unclear whether children with ASD have reduced range of motion or simply have a more rigid gait pattern than healthy controls. This would fit in with the aforementioned idea that children with ASD seek to stabilise their gait. Further research in the area should aim to clarify this.

Only two studies investigated kinetic gait parameters and yielded few results of significance. The only significant differences in joint moments between children with ASD and healthy controls were in peak ankle plantar flexion moments and peak hip flexor moments. The children with ASD had reduced peak plantar flexion moments, meaning that the forces acting around the ankle joint during flexion were reduced when compared to controls. Since children with ASD have been shown to have reduced plantar flexion at toe-off [41], it makes sense that the forces generated would also be reduced [41]. This finding may imply a weakness of the plantar flexor muscles or may be due to the group's reduced peak plantar flexion angles as less force is required to generate a smaller movement in the joint. The author suggested that it may also be caused by hypotonia, which was confirmed in one-third of the children diagnosed with ASD. Children with ASD were also found to have reduced peak hip flexor moments but had increased hip flexion angles. This may imply weakness in the hip flexor muscles as they are unable to generate the same amount of force as those that healthy controls can, and the increased angles may imply weakness or a lack of control of the hip flexor muscles.

The findings of many studies in children with ASD concluded that gait abnormalities observed are indicative of widespread dysfunction in cerebellar and frontostriatal basal ganglia circuitry [28, 32, 33, 35, 37–41]. This is in agreement with Hallett et al., who found that adults with ASD had a gait pattern similar to patients with Parkinson's disease and suggested cerebellar involvement. A study compared brain images of children with ASD to healthy controls and showed abnormal cerebellar maturation in the ASD group [44]. The kinematic gait pattern exhibited by children with ASD shares common characteristics with “crouch gait,” a pattern commonly elicited in Parkinson's disorder which involves changes to the cerebellum. As referenced previously a wide range of studies point towards cerebellar deficits in ASD based on postmortem histopathological studies, structural and functional imaging. It has been argued that cerebellar dysfunction may explain the heterogeneous deficits observed in ASD, both sensory-motor and cognitive [45].

However, the increased variability of gait parameters exhibited by children with ASD, as examined by three studies, may suggest an association with extensive neurobiological dysfunction which is unlike adult-onset disorders such as Parkinson's disorder [28, 35, 38]. Furthermore, the lack of improvement with visual cues as well as increase in variability of gait parameters with dual-task observed in children with ASD lends strength to the argument for ASD to be viewed as a “disorder of complex information processing” as initially proposed by Minshew and Goldstein [46].

Velocity has been shown to affect gait patterns [47–49]. No study examining gait in children with ASD has controlled for velocity, which may lead to velocity being a confounding factor. Although no study found velocity to be significantly reduced in children with ASD, this does not mean that each child walked at the same velocity. It is very difficult to impose a standard velocity across groups but this may lead to deviations in gait patterns becoming more apparent.

There are also study-design considerations in relation to the studies reviewed here. Sample sizes in all studies were quite small with the cohort studied by Shetreat-Klein et al. [32] being by far the largest at 76 including 38 children diagnosed with ASD. By definition, ASD is a spectrum rather than one specific condition with differing subtypes and varying levels of severity. These high levels of variability in this group, combined with small sample groups, may lead to between-group differences to be obscured. Several studies cited this as a limitation to their research and recommended that future studies include a larger cohort [34–36, 40].

Furthermore ASDs frequently are accompanied by a range of comorbid conditions such as attention deficit hyperactivity disorder (ADHD), developmental coordination disorder (DCD), and anxiety disorders. The* Fifth Edition of the Diagnostic and Statistical Manual of Mental Disorders* [2] by the American Psychiatric Association now permits the diagnosis of comorbid ADHD and DCD with ASD. The extent to which gait abnormalities in ASD are unique or associated with other comorbid conditions remains unclear and existing studies have not addressed this issue. This is a question therefore for future study.

Overall, the studies reviewed had significant differences in terms of methodology, thus reducing comparability of their results. This may explain some of the inconsistencies found; for example, Vilensky et al. [41] found that the ASD group had increased time in stance phase whereas Rinehart et al. [28] found no significant difference in the same parameter, the split opinion on stride/step length. These may be accounted for by the differing inclusion criteria such as age: the mean age of the children with ASD in the study by Vilensky et al. [41] was 6.1 years, with the youngest being just three, compared to the group studied by Rinehart et al. [28] which had a mean age of 10.7 years and the youngest was six years old. Gait patterns develop as a child grows so patterns will be different with differing age ranges, making it very difficult to compare the gait patterns observed. There were also differences in IQ between the groups of both studies, with Rinehart et al. [28] only including “normally intelligent” children, but Vilensky et al. [41] had seven children who were classed as “severely retarded” which may influence movement patterns, and the two studies used different gait analysis systems so differences in detection of patterns may arise.

Many children with ASD are prescribed antipsychotic medications, which may have effects on certain parts of the brain responsible for the control of movement such as the cerebellum. Only Nobile et al. [37] specified that all the children included in the study were drug naïve. Nayate et al. [35] cited this as a limitation to their research as three children in the study were on a mood-altering drug called sodium valproate which, at high doses, may have clinical effects on the cerebellum [50], which in turn may affect gross motor control and, therefore, movement patterns.

The temporospatial patterns exhibited by children with ASD were similar to the gait of children with obesity, with a wide base of support and shorter strides [51]. None of the studies reviewed examined the effects of BMI or body weight as a confounding factor. To date, no study has been published which compares gait patterns in children with ASD to healthy controls and also to children with obesity. It may also be of clinical importance to compare gait patterns in children with ASD to children with obesity as it has been shown that children with ASD have, by temporospatial parameters, a similar gait to children with obesity and have, on average, a significantly higher body fat percentage and lower lean tissue masses than healthy controls [52].

There are several factors that may affect research in this area such as intellectual ability, behavioural problems, and severity of the condition. Most studies in the area examined gait patterns in children with high-functioning ASD. This means that the current research cannot provide an overall picture of gait deviations in children across the autism spectrum disorders. The question of whether changes in gait patterns become more obvious with increasing severity of the disorder was raised by Weiss et al. [33]. They suggested that future research should be carried out including more low-functioning individuals.

The studies reviewed are helping to provide clinical practitioners, across a variety of disciplines, with a description of physical characteristics of ASD. The knowledge and understanding of this aspect of ASD may increase routine referral to services, such as physiotherapy, and allow for better intervention and treatment planning. Since children with ASD were found to have reduced range of motion during gait [37, 40, 41], there may be underlying weakness within muscles of the lower limb and the wider base of support found [32, 35, 37, 40] may imply issues with balance and/or proprioception. An assessment-based individually tailored exercise programme including gait reeducation, lower limb strengthening and balance, and proprioceptive training may improve gait patterns and coordination, with the overall aim of increasing physical activity and quality of life for children with ASD.

## 5. Conclusion

In conclusion, the overall findings of the studies conducted in the area are inconclusive, due to a number of confounding factors as discussed; however, some results suggest an emerging pattern. The current perspective on gait patterns in children with ASD is that there are a number of deviations present in terms of temporospatial, kinematic, and kinetic parameters and that gait, along with other movement pattern changes, may be used to allow for earlier diagnosis of ASD. There is, however, some consensus regarding the involvement of the cerebellum and basal ganglia in children with ASD and the relationship with observed motor deficits. Several limitations have been acknowledged and future research will need to address these more rigorously. More research should be done comparing children with ASD to other diagnostic groups to determine the degree of specificity of deficits and whether observed deficits influence treatment planning.

## Figures and Tables

**Table 1:** Studies included in review.

Author	Year	Title	Sample size total (ASD)	Average age in sample (years)	Gait analysis method	Major findings
Shetreat-Klein et al. [32]	2014	Abnormalities of joint mobility and gait in children with autism spectrum disorders	76 (38)	4.58	Video Analysis	Gait with wide base of support common in ASD

Weiss et al. [33]	2013	Gait analysis of teenagers and young adults diagnosed with autism and severe verbal communication disorders	19 (9)	19	GAITRite	Reduced stride length and increased stance time in ASD

Chester and Calhoun [34]	2012	Gait symmetry in children with autism	36 (14)	6.06	8-Cam Vicon	No significant differences in mean temporospatial gait parameters

Nayate et al. [35]	2012	Differentiation of high-functioning autism and Asperger's disorder based on neuromotor behaviour	33 (11)	12.75	GAITRite	Increased step width in ASD; visual cues increased stride length variability in ASD

Calhoun et al. [36]	2011	Gait patterns in children with autism	34 (12)	6.06	8-Cam Vicon	Increased cadence, reduced peak ankle plantar flexion and hip flexion moments in ASD

Nobile et al. [37]	2011	Further evidence of complex motor dysfunction in drug naive children with autism using automatic motion analysis of gait	32 (16)	10.28	ELITE	Increased step width, reduced ankle plantar flexion and knee flexion-extension at toe-off, and a reduced hip range of motion in ASD

Rinehart et al. (a) [28]	2006	Gait function in high-functioning autism and Asperger's disorder: evidence for basal-ganglia and cerebellar involvement?	30 (10)	10.69	Clinical Stride Analyzer	Increased variability in stride length in ASD

Rinehart et al. (b) [38]	2006	Gait function in newly diagnosed children with autism: cerebellar and basal ganglia related motor disorder	22 (11)	5.79	GAITRite	Increased variability in stride length and stride time in ASD

Vernazza-Martin et al. [39]	2005	Goal directed locomotion and balance control in autistic children	15 (9)	5	ELITE	Reduced step length in ASD

Ambrosini et al. [40]	1998	Motion analysis of patients with infantile autism	8 (8)	10.8	5 Cam Vicon	Reduced stride length, increased step width, and reduced ground reaction forces during terminal stance in ASD

Vilensky et al. [41]	1981	Gait disturbances in patients with autistic behavior: a preliminary study	41 (21)	7.73	Video Analysis	Reduced stride length and increased stance time in ASD. Reduced ankle dorsiflexion and knee extension at initial contact and increased hip flexion at toe-off in ASD

## Abbreviation

ASD: Autism spectrum disorder.
